# Scotch Physicians and Surgeons

**Published:** 1888-01-21

**Authors:** C. G. Furley


### Scotch Physicians and Surgeons.

By C. G. Furley.

(*Continued from p. 247.*)

### SIR JAMES Y. SIMPSON.

While the storm was still raging around the vacant chair Simpson married, on December 29th, 1839. Whether or not this helped his cause, it so befell that on February 4th, 1840, he was elected to the professorship. The success of his candidature delighted him; his letters to his own relatives and his wife's show how intense had been his anxiety to obtain the chair; but the change in his position was not at first without counterbalancing disadvantages. He had been poor before, but it was during the first months of his professorship that he felt most severely the strain of poverty. A married man and a University professor cannot live in quite the same style as an irresponsible bachelor practitioner; and his practice, though extensive and extending, was still of a kind that brought him more distinction than money. Moreover, he could not then push himself into remunerative work, for he had to prepare the lectures he meant to deliver during the ensuing winter; while, at the same time, he felt obliged by his new status to give up the delivery of a course of extra-mural lectures which he had planned. This time of anxiety and embarrassment was, however, brief. The first session of his professorship relieved him from further cause of care, the fees amounting to £600; and within three years fame and fortune were striding on hand in hand.

It was inevitable, human nature being what it is—its angelic qualities somewhat overweighted by less attractive characteristics derived from our simian ancestors—that so swift a rise should lead to professional jealousy; and Simpson's nature was not wholly of a kind to disarm the jealousy of less successful men. He was too blunt, too outspoken, to avoid giving offence; and already the charge, pushed more strenuously in later days, that he ignored the requirements and reserve of professional etiquette, was brought against him. Ill-natured things were said and written of him, and, in return, he said and wrote some things which were certainly ill-advised. "Have you seen the atrocious, lying article about me in such a paper?" he asked an acquaintance, who happened to be the author of the article in question. After such an unlucky shot as this it was natural that intercourse should be reduced to the coldest and narrowest exchange of civilities. Again, he wrote to the writer of a very sharp attack on him in this wise:

"Dear Dr. ——, I have read your very absurd and indecent attack on me. If you will only come and spend a few days with me (you will always find a knife and fork ready, and a good Scotch welcome), I will undertake to convince you of your error in forty-eight hours."

No doubt Simpson thought this was a kind, friendly, and forgiving letter. What Dr.—— thought of it is not recorded.

Meanwhile, in spite of all things, Simpson prospered. His figure—the "body of Bacchus with the head of Jove," as Gerald aptly describes it—was pointed out to visitors to Edinburgh as that of one of its celebrities. He was sent for to great distances to attend difficult cases or give advice on problems of gynæcology, and the height of fame—or, at least, notoriety—may be held to have been reached when, on his being called to London to attend a member of the Duke of Sutherland's family, he found that a "life" of him had been published on the day of his arrival.

Patients of all ranks flocked to him. He saw those whose cases seemed to him most serious, and let the others go. This behaviour created some irritation. A wealthy hysterical lady, whose chief complaint is idleness, feels ill-used if she is neglected for the sake of a ploughman's wife. It may be that there were some cases of genuine neglect. It is hardly possible that any one man could give as much attention as she desired to each member of such a crowd as Sir Robert Christison depicts besieging Dr. Simpson's house in Queen Street. The dining-room, as well as the waiting-room, was full of ladies; female faces looked out from the windows of the hall and staircase, and as Dr. Christison came up a lady was ringing the door-bell. But the negligence was more apparent than real. When a physician sees at a glance what is the matter with a patient, he cannot, if he has fifty other patients waiting for him, waste time in listening to what she thinks is the cause of her disease, or what Drs. A, B, and C, whom she has successively consulted, opine as to its nature. Simpson had too much to do in the diagnosis and cure of the most serious and agonising diseases that afflict womanhood to have time to spare for those needless, though pleasant, courtesies which are most valued by those who ail least.

Nevertheless his lack of an exact method of keeping a list of his patients led to forgetfulness and unpunctuality, the besetting sins of medical men. Patients became irritated, and sought other advice, perhaps from some amiable fellow-practitioner, who mentioned casually, "You are the third lady who has come to me in consequence of Dr. Simpson's carelessness." If he were interrupted in a case of importance by a call to a attend to a new patient he would promise a visit "to-morrow;" but the engagement not being noted, and the morrow bringing its own occupations and interests, the promise was forgotten, and hence arose complaints, and rumours of neglect that had occasionally led to fatal result. And yet in spite of all this fault-finding patients continued to come, and in increasing numbers.

The same carelessness marked his conduct in money matters, once his position was established, and the need of such strict economy as marked his early days had vanished. Fees were not acknowledged on their receipt, and those who sent them, especially those whose own lives were so idle that they could not realise how busy the doctor's was, felt annoyed and neglected. Dr. Duns, Simpson's biographer, tells an amusing story of the fate of a ten-pound note:

"One man of wealth sent £10 in circumstances in which £100 might have been expected, adding, 'I am not sure if the amount is more or less than is usual on such occasions, but I will be guided by what you say when you acknowledge this.' It was not acknowledged, and note after note arrived in fevered haste: 'You have not acknowledged remittance of 12th, of £10. I hope it reached you.' Later, 'There must surely be some mistake.' And later still, 'Your conduct is surprising,' etc. But in vain. Perhaps the writer would have been more indignant still had he known the treatment his fee received. It was put carelessly into a capacious pocket, which received and held, often for a week or two, many strange epistles. During a boisterous night Simpson's sleep was disturbed by the rattling of the window-frame, and drawing on his pocket for a bit of paper, wherewith to tighten the loose sash, the £10 note had come readiest to hand, and was employed for this purpose. It was found by Mrs. Simpson in the morning in this useful position. She showed it to her husband, doubtless with a grave and reproachful face; for women, whose life-work deals with the small economies of the household, have naturally a greater reverence for money than is possessed by men. But Simpson's only remark was 'Oh! it's *that* £10.'"

In fact, money "burnt a hole in his pocket," as nurses say to children. In his later life some people said he was avaricious, because he embarked in various speculations, many of which turned out unprofitably. But the truth was that he could not leave money to lie still and accumulate in the orthodox grooves; he preferred even a rash investment to that. On the other hand, his genorosity to others was almost reckless. No one knows how many students' class fees he remitted, or how many patients' fees he returned; but these are the common generosities of almost every doctor and professor. But when young practitioners, struggling with the inevitable expenses of a first year's work, before bills begin to be paid, appealed to him for help, they were rarely refused, even though his personal acquaintance with them was of the slightest. He remembered his own first steps, when but for his brother's help he could not have "warstled through" the troubles and anxieties that beset the beginning of a career; and gave to others the help he had required himself. Others, less worthy probably than these, appealed to him successfully—antiquaries of all grades, trading upon his archæological tastes, and visionaries of every kind. One belated alchemist, seeking for the philosopher's stone, asked and obtained money to help him in the prosecution of his experiments. Not that Simpson's clear, scientific brain cherished the faintest belief in the mystic stone that could transmute the baser metals into gold, but —the man was poor.

The year 1847 was a memorable one, perhaps the most memorable in Simpson's life. In January he was appointed "one of her Majesty's physicians for Scotland." The appointment gratified him as a public testimony to his merits, and was made more valuable by the Queen's statement of the satisfaction with which she made it. He expressed the pleasure he felt in a letter to the loyal brother at Bathgate; but he adds: "Flattery from the Queen is perhaps not common flattery, but I am far less interested in it than in having delivered a woman this week, without any pain, while inhaling sulphuric ether."

At this time he was greatly interested in the action of ether, and advised many of his friends to use it. Liston, the great surgeon, had employed it successfully in many operations, and Simpson became convinced that it would be serviceable to the accoucheur. But the results were not satisfactory. The anæsthesia produced by ether was not sufficiently complete and permanent for all purposes, and the sense of choking in the inhalation, as well as the occasionally alarming after-effects, offered objections to its regular use. Simpson's active mind, therefore, went in search of some more satisfactory anæsthetic. He made various experiments, and discussed the matter with several friends, among them one Mr. Waldie, an old West Lothian friend who had settled in Liverpool as a chemist. It was in the course of a conversation with him that Simpson's attention was directed to chloroform; and as the result of the experiments he made with it, came that paper read before the Medico-Chirurgical Society of Edinburgh, which told the glad tidings of a new, safe, and lasting anæsthetic.

*(To be continued.)*

